# Supplementary material for: Zic-HILIC MS/MS Method for NADomics Provides Novel Insights into Redox Homeostasis in Escherichia coli BL21 Under Microaerobic and Anaerobic Conditions
Source: Metabolites. 2024 Nov 9;14(11):607. doi: 10.3390/metabo14110607 (PMC11596675; doi:10.3390/metabo14110607)
Supplement: Supplementary file 1 [file metabolites-14-00607-s001.zip › metabolites-3248822-supplementary_v1/Supplementary figure S1.pdf]

Supplementary Figure S1. (a) A chromatogram of NAAD MRM transition of 100 nM standard sample (without matrix) showing baseline separation between NAD^+ and NAAD and NAD^+ bleed through in NAAD transition (b) A chromatogram of NAAD MRM transition of 100 nM standard sample (spiked with matrix) showing no detectable peak for NAAD and bleed through of NAD^+ from the matrix in NAAD transition (c) A chromatogram of NAAD MRM transition in *E.coli* BL 21 sample extract showing no detectable peak for NAAD and NAD^+ bleed through.

(a)

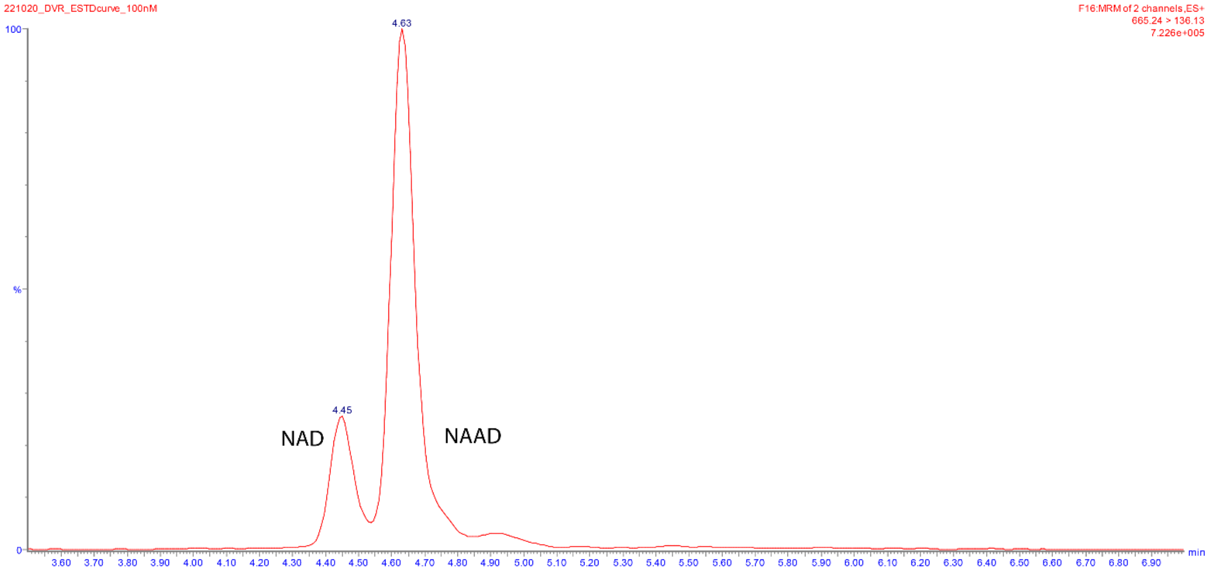

(b)

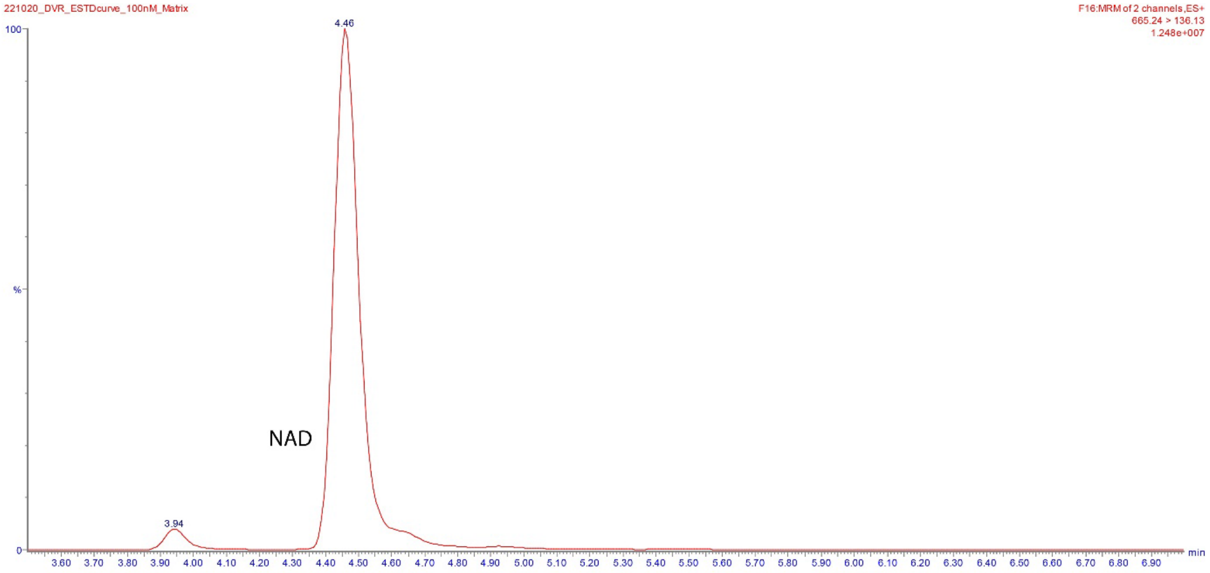

(c)

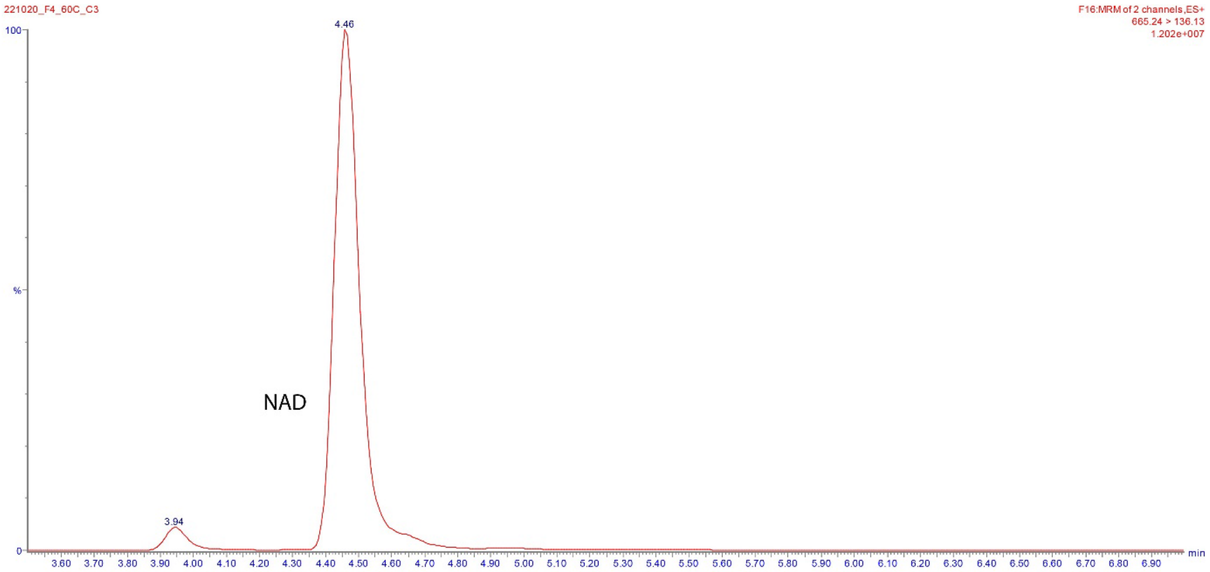

221020_F4_60C_C3
F16:MRM of 2 channels,ES+
665.24 > 136.13
1.202e+007
100
%
0
4.46
3.94
NAD
3.60
3.70
3.80
3.90
4.00
4.10
4.20
4.30
4.40
4.50
4.60
4.70
4.80
4.90
5.00
5.10
5.20
5.30
5.40
5.50
5.60
5.70
5.80
5.90
6.00
6.10
6.20
6.30
6.40
6.50
6.60
6.70
6.80
6.90
min
