# Supplementary material for: Zic-HILIC MS/MS Method for NADomics Provides Novel Insights into Redox Homeostasis in Escherichia coli BL21 Under Microaerobic and Anaerobic Conditions
Source: Metabolites. 2024 Nov 9;14(11):607. doi: 10.3390/metabo14110607 (PMC11596675; doi:10.3390/metabo14110607)
Supplement: Supplementary file 1 [file metabolites-14-00607-s001.zip › metabolites-3248822-supplementary_v1/Supplementary figure S2.pdf]

Supplementary Figure S2. Chromatographic peak shapes and resolution (a) using extraction solvent ACN:MeOH: H2O, 15 mM ammonium acetate pH 9.7 ((60:20:20); (b) using modified extraction solvent ACN:MeOH: H2O, 15 mM ammonium acetate pH 9.7 ((55:20:25)

(a)

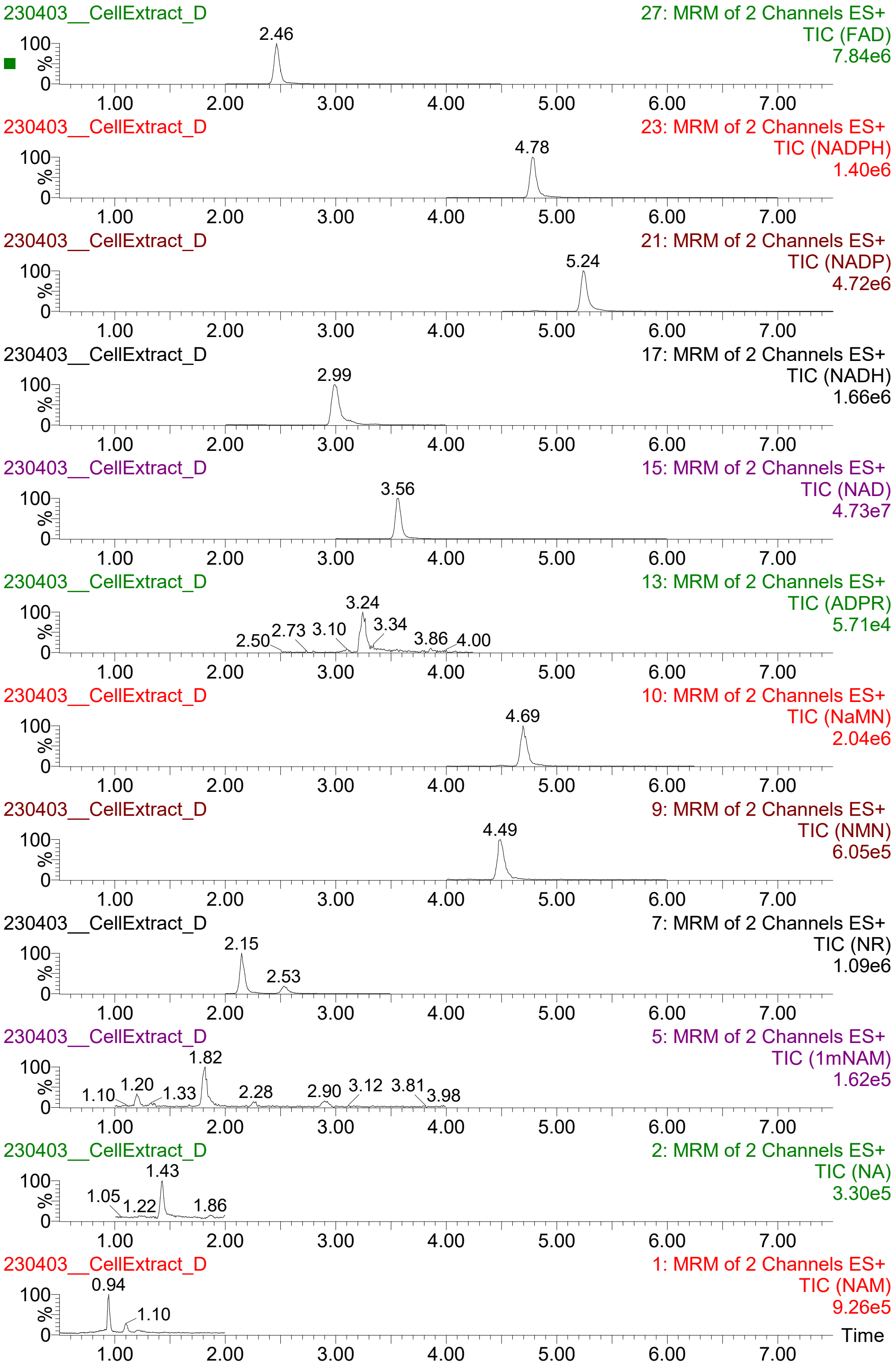

(b)

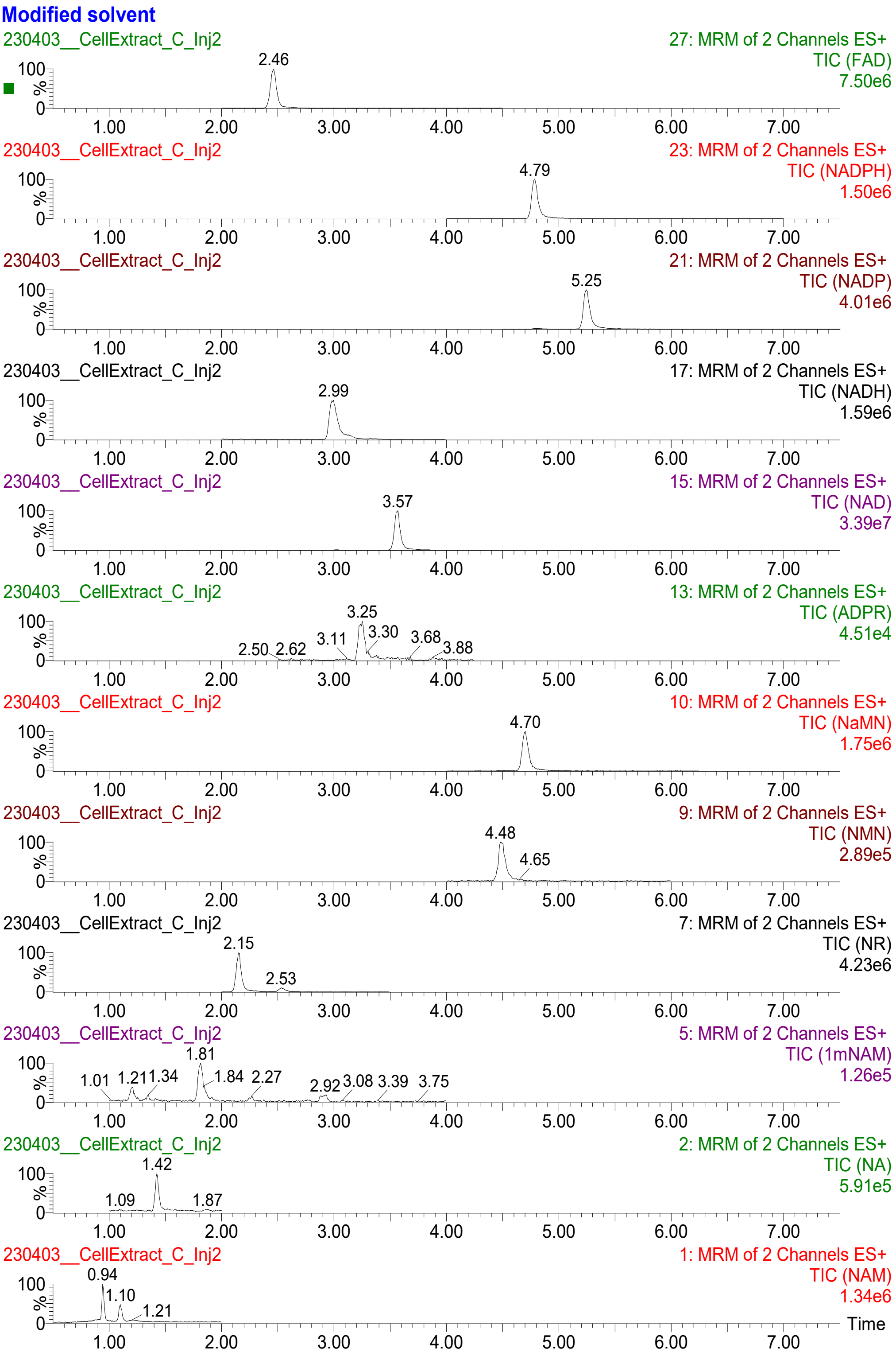

NA in chromatograms stands for NCA (Nicotinic acid).
