# Supplementary material for: Zic-HILIC MS/MS Method for NADomics Provides Novel Insights into Redox Homeostasis in Escherichia coli BL21 Under Microaerobic and Anaerobic Conditions
Source: Metabolites. 2024 Nov 9;14(11):607. doi: 10.3390/metabo14110607 (PMC11596675; doi:10.3390/metabo14110607)

Supplementary Figure S3. Cultivation of *E. coli* BL21 in aerobic conditions with DO ≥ 40% maintained by DO-agitation cascade. The specific growth rate (μ) was observed to be (0.64 ± 0.01) h^{-1} throughout the exponential growth phase.

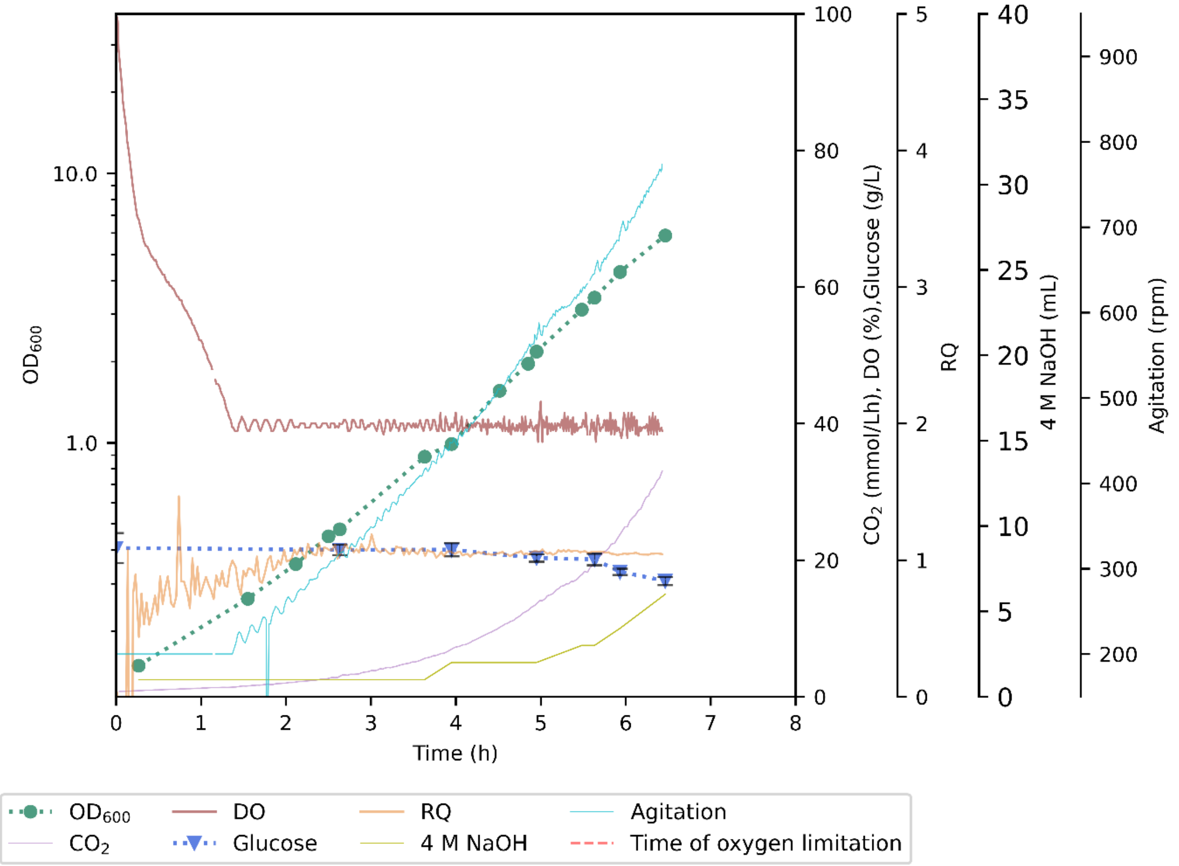

Supplement: Supplementary file 1 [file metabolites-14-00607-s001.zip › metabolites-3248822-supplementary_v1/Supplementary figure S3.pdf]
