# Supplementary material for: Zic-HILIC MS/MS Method for NADomics Provides Novel Insights into Redox Homeostasis in Escherichia coli BL21 Under Microaerobic and Anaerobic Conditions
Source: Metabolites. 2024 Nov 9;14(11):607. doi: 10.3390/metabo14110607 (PMC11596675; doi:10.3390/metabo14110607)
Supplement: Supplementary file 1 [file metabolites-14-00607-s001.zip › metabolites-3248822-supplementary_v1/Supplementary table S1.pdf]

Supplementary Table S1. MRM parameters for ^{13}C isotopologues used for isotope dilution for metabolites listed in Table 1

Compound Name	Parent ion (m/z)	Transition for quantification (m/z)	Cone Voltage (V)	Collision Energy (eV)
NAM ^{13}C	129.1	84.8	26.0	22.0
NR ^{13}C	266.9	129.7	14.0	8.0
FAD ^{13}C	813.2	358.0	20.0	22.0
NADH ^{13}C	687.1	670.0	20.0	17.0
NAD^{+} ^{13}C	685.1	438.0	20.0	26.0
NAMN ^{13}C	347.0	130.1	22.0	12.0
NADPH ^{13}C	767.1	750.0	20.0	17.0
$NADP^{+}$ ^{13}C	765.1	619.0	20.0	20.0
