# Supplementary material for: Zic-HILIC MS/MS Method for NADomics Provides Novel Insights into Redox Homeostasis in Escherichia coli BL21 Under Microaerobic and Anaerobic Conditions
Source: Metabolites. 2024 Nov 9;14(11):607. doi: 10.3390/metabo14110607 (PMC11596675; doi:10.3390/metabo14110607)
Supplement: Supplementary file 1 [file metabolites-14-00607-s001.zip › metabolites-3248822-supplementary_v1/Supplementary table S2.pdf]

Supplementary Table S2. Absolute metabolite concentrations (nmol g^{-1} CDW) in *E.coli* BL21 extracted using different ratios of extraction solvents for quantification using the zic-HILIC MS/MS method. The table indicates average values from technical replicates (n=3), standard deviation (SD), and relative standard deviation (RSD,%) for each metabolite. No peak was observed for 1-mNAM. Outliers were removed using Dixon's Q tests with a 95% confidence level [31]

Extraction Solvent		NAM	NCA	NR	FAD	NADH	ADPR	NAD^+	NMN	NAMN	NADPH	$NADP^+$
	Average	4.6	3.4	1.4	66.4	252.7	1.6	747.8	4.4	19.4	51.6	152.5
ACN: MeOH: H_2O, 15 mM NH_4OAc pH 9.7 (60:20:20)	SD	1.3	0.3	0.3	0.7	18.3	0.2	41.1	0.3	2.7	2.9	24.4
	RSD	27.8	10.4	20.2	1.0	7.2	14.3	5.5	7.4	14.0	5.7	16.0
	Average	6.1	7.7	3.0	74.0	265.3	1.5	769.7	2.9	24.5	62.4	143.9
ACN: MeOH: H_2O, 15 mM NH_4OAc pH 9.7 (55:20:25)	SD	1.8	1.2	0.1	5.6	14.5	0.1	79.6	0.3	2.3	3.5	16.2
	RSD	29.1	15.2	2.2	7.6	5.5	7.3	10.3	9.7	9.3	5.7	11.2
