# Supplementary material for: Zic-HILIC MS/MS Method for NADomics Provides Novel Insights into Redox Homeostasis in Escherichia coli BL21 Under Microaerobic and Anaerobic Conditions
Source: Metabolites. 2024 Nov 9;14(11):607. doi: 10.3390/metabo14110607 (PMC11596675; doi:10.3390/metabo14110607)
Supplement: Supplementary file 1 [file metabolites-14-00607-s001.zip › metabolites-3248822-supplementary_v1/Supplementary table S3.pdf]

Supplementary Table S3. Intracellular absolute concentrations (nmol g^{-1} CDW) in *E.coli* BL21 extracted at different temperatures for quantification using zic-HILIC MS/MS method. The table indicates average values from technical replicates (n=5), standard deviation (SD), and relative standard deviation (RSD,%) for each metabolite. No peak observed for 1-mNAM. Outliers were removed using Dixon's Q tests with a 95% confidence level [31].

Temperature		NAM	NCA	NR	FAD	NADH	ADPR	NAD$^+$	NMN	NAMN	NADPH	NADP$^+$
						Preliminary studies						
4°C	Average	8.4	11.7	14.6	48.5	174.1	-*	235.1	20.0	17.9	23.3	7.9
	SD	0.6	1.3	1.9	6.8	19.7		35.0	1.8	1.3	2.8	1.5
	RSD	7.2	10.8	12.9	14.0	11.3		14.9	8.9	7.0	12.2	18.9
20°C	Average	18.6	15.6	8.2	59.0	179.3		274.7	21.1	18.1	27.1	8.7
	SD	11.1	10.8	1.9	3.9	3.6		18.7	1.6	1.3	4.0	0.8
	RSD	59.9	69.0	22.9	6.6	2.0		6.8	7.6	7.4	14.6	9.2
60°C	Average	8.8	10.7	8.1	103.1	147.4		326.7	8.9	21.3	26.7	10.0
	SD	0.7	0.8	1.3	8.7	7.1		24.0	0.3	1.8	2.9	1.0
	RSD	7.9	7.7	16.1	8.4	4.8		7.4	3.1	8.7	10.7	10.3
80°C	Average	6.5	8.5	5.2	109.1	126.2		320.8	3.3	15.4	27.1	9.4
	SD	1.0	8.3	5.1	8.2	6.9		23.5	0.0	0.7	3.0	1.0
	RSD	16.1	97.3	98.8	7.5	5.4		7.3	0.6	4.7	11.2	10.5

Temperature		NAM	NCA	NR	FAD	NADH	ADPR	NAD+	NMN	NAMN	NADPH	NADP+
		Final study										
20°C	Average	4.9	42.3	21.1	51.6	384.8	7.7	126.9	30.6	23.9	30.4	1.8
	SD	1.5	6.7	2.7	12.4	24.4	0.1	32.5	8.0	3.1	4.5	0.5
	RSD	31.5	15.8	12.7	24.1	6.3	1.3	25.6	26.3	13.0	14.8	25.2
40°C	Average	4.2	16.1	13.9	80.4	375.7	7.9	150.7	15.0	35.6	33.0	2.3
	SD	1.3	4.1	5.0	11.2	39.1	0.1	27.1	2.5	5.9	8.3	0.5
	RSD	32.2	25.2	35.7	14.0	10.4	1.5	18.0	16.4	16.6	25.1	20.8
60°C	Average	6.6	19.8	7.0	95.8	299.8	7.9	154.2	2.7	44.8	28.6	2.2
	SD	2.4	8.8	2.8	13.1	18.1	0.1	25.1	1.1	7.6	2.6	0.4
	RSD	36.2	44.7	39.2	13.7	6.0	1.4	16.3	39.9	17.0	9.3	17.8

*: ADPR was not included in the preliminary study.
