# Supplementary material for: Zic-HILIC MS/MS Method for NADomics Provides Novel Insights into Redox Homeostasis in Escherichia coli BL21 Under Microaerobic and Anaerobic Conditions
Source: Metabolites. 2024 Nov 9;14(11):607. doi: 10.3390/metabo14110607 (PMC11596675; doi:10.3390/metabo14110607)
Supplement: Supplementary file 1 [file metabolites-14-00607-s001.zip › metabolites-3248822-supplementary_v1/Supplementary table S4.pdf]

Supplementary Table S4. Recovery (%) obtained after freeze–drying of QC (quality control) standards with concentrations, LQC: 250nM, MQC: 2500 nM, and HQC:7500 nM, expressed as an average of recoveries obtained from technical replicates ± SD (standard deviation).

**Metabolite**	**% Recovery**		
	**LQC**	**MQC**	**HQC**
NAM	115.4 ± 7.5	125.2 ± 14.2	106.5 ± 2.4
NCA	122.5 ± 6.5	124.2± 13.7	103 ± 2.8
1-mNAM	91.1 ± 14.1	90.4 ± 9	121.5 ± 5.9
NR	91.20 ± 12.8	91.6 ± 10.5	104.3 ± 5.8
FAD	74.8± 6.3	96.4 ± 3.3	104.0 ± 4.5
NADH	8.6 ± 2.4	15.4 ± 3.7	21.8 ± 1.02
ADPR	97.3 ± 13.6	143.2 ± 35.4	134.4 ± 16.0
NAD^+	92. 1± 2.9	84.6 ± 3.3	84.6 ± 4.9
NMN	84.1 ± 8.6	82.3 ± 11.7	103.3 ± 13.5
NAMN	110.6 ± 20.5	85.9 ± 12.2	131.8 ± 9.3
NADPH	21.6 ± 3.5	26.5 ± 4.8	33 ± 1.4
$NADP^+$	102.1 ± 8.6	111.5 ± 9.6	113.2 ± 1.0
