# Supplementary material for: Zic-HILIC MS/MS Method for NADomics Provides Novel Insights into Redox Homeostasis in Escherichia coli BL21 Under Microaerobic and Anaerobic Conditions
Source: Metabolites. 2024 Nov 9;14(11):607. doi: 10.3390/metabo14110607 (PMC11596675; doi:10.3390/metabo14110607)
Supplement: Supplementary file 1 [file metabolites-14-00607-s001.zip › metabolites-3248822-supplementary_v1/Supplementary table S5.pdf]

Supplementary Table S5. Intraday and inter-day precision (determined by the coefficient of variation (CV, %)) and accuracy(determined by calculating relative bias (bias,%)) for the metabolites at three standard concentrations, spiked with 90% v/v matrix (LQC: 250 nM; MQC: 2500 nM and HQC: 7500 nM).

Metabolite	Precision						Accuracy					
	CV*, % (intraday)			CV*, % (Interday)			Relative bias**, % (Intraday)			Relative bias**, % (Interday)		
	LQC	MQC	HQC	LQC	MQC	HQC	LQC	MQC	HQC	LQC	MQC	HQC
NAM	1.6	3.2	5.6	7.7	9.6	13.0	13.3	18.0	20.3	11.5	-11.0	-7.1
NCA	6.2	5.7	4.3	12.4	18.0	24.8	-3.6	-6.1	-4.8	-5.2	-25.0	-22.9
1m-NAM	7.0	4.0	0.6	10.8	4.2	4.2	2.5	-5.2	-2.2	20.5	-0.1	-5.2
NR	5.6	4.9	1.8	4.5	1.1	3.0	-4.6	-5.2	0.7	-5.1	0.3	4.3
FAD	0.6	1.3	0.4	5.5	4.0	2.9	11.1	-12.9	-7.5	8.2	-10.5	-0.1
NADH	3.6	1.9	2.8	1.6	2.0	7.8	-	11.6	15.0	-	0.3	13.1
ADPR	19.7	7.6	16.6	15.2	17.4	11.1	-16.6	-0.3	-11.3	-4.6	0.5	0.6
NAD^+	13.7	6.2	5.5	9.7	2.6	5.3	-	32.3	4.0	-	12.8	2.5
NMN	4.0	3.7	18.6	2.9	7.0	2.1	-8.9	-7.5	13.3	-10.2	-3.1	0.3
NAMN	4.5	4.5	2.1	2.9	3.2	5.1	1.5	2.6	1.9	-0.5	-2.1	3.6
NADPH	6.7	7.9	7.9	9.3	5.5	5.9	8.9	-2.5	1.7	-12.2	-6.1	11.2
$NADP^+$	3.4	2.1	1.3	5.7	4.6	3.8	20.6	-7.9	-4.3	18.5	6.4	3.9

* CV, % = (Standard deviation/ Mean) x 100

**Relative bias, % = (Mean calculated concentration – Theoretical concentration)/Theoretical concentration x 100
