# Supplementary material for: Zic-HILIC MS/MS Method for NADomics Provides Novel Insights into Redox Homeostasis in Escherichia coli BL21 Under Microaerobic and Anaerobic Conditions
Source: Metabolites. 2024 Nov 9;14(11):607. doi: 10.3390/metabo14110607 (PMC11596675; doi:10.3390/metabo14110607)
Supplement: Supplementary file 1 [file metabolites-14-00607-s001.zip › metabolites-3248822-supplementary_v1/Supplementary table S6.pdf]

Supplementary Table S6. Stability of metabolites in external standard solutions (LQC: 250 nM; MQC: 2500 nM and HQC: 7500 nM), spiked with 90% v/v matrix, kept in autosampler for 24h. Percentage recovery for a particular metabolite was calculated by dividing the MS response of a QC sample of interest (incubated 24h in an autosampler or a freeze-thawed sample) by the MS response of the same sample at a zero hour (at the start of incubation) and is expressed as an average recovery from technical replicates ± SD (standard deviation).

**Metabolite**	**Recovery (%)**		
	**LQC**	**MQC**	**HQC**
NAM	85.5 ± 0.7	79.7 ± 2.3	74.9 ± 0.9
NCA	82.0 ± 2.6	77.5 ± 0.9	75.1 ±0.3
1mNAM	103.6 ± 10.2	99.2 ± 4.2	96.5 ± 8.9
NR	105.0 ± 3.2	108.6 ± 2.6	104.5 ± 5.4
FAD	108.0 ± 1.04	108.4 ± 6.4	114.6 ± 5.5
NADH	103.4 ± 4.5	109.8 ± 1.6	101.9 ± 3.2
ADPR	130.1 ± 6.7	136.7 ± 17.8	120.9 ± 10.3
NAD^+	115.4 ± 5.7	108.2 ± 3.2	110.8 ± 8.4
NMN	99.6 ± 3	108.3 ± 1.9	86.3 ± 0.3
NAMN	96.3 ± 0.4	95.0 ± 0.2	94.7 ± 0.3
NADPH	89.4 ± 7.9	98.5 ± 1.5	102.2 ± 5.4
$NADP^+$	99.6 ± 0.3	105.9 ± 3.4	98.8 ± 5.8
