# Supplementary material for: Zic-HILIC MS/MS Method for NADomics Provides Novel Insights into Redox Homeostasis in Escherichia coli BL21 Under Microaerobic and Anaerobic Conditions
Source: Metabolites. 2024 Nov 9;14(11):607. doi: 10.3390/metabo14110607 (PMC11596675; doi:10.3390/metabo14110607)
Supplement: Supplementary file 1 [file metabolites-14-00607-s001.zip › metabolites-3248822-supplementary_v1/Supplementary table S7.pdf]

Supplementary Table S7. Stability of metabolites in *E.coli* cell extract and external standard solutions (LLOQ=78.1 nM, LQC=312.5 nM, MQC= 2500 nM, and HQC=7500 nM), spiked with 90% v/v matrix, through three consecutive freeze–thaw cycles, expressed as average recovery (%) of technical replicates ± SD. The recovery (%) was calculated by dividing the response after each consecutive freeze–thaw cycle by the response obtained before freezing the samples.

QC Sample	Recovery (%)											
	**NAM**	**NCA**	**1mNAM**	**NR**	**FAD**	**NADH**	**ADPR**	**NAD$^+$**	**NMN**	**NAMN**	**NADPH**	**NADP$^+$**
**Freeze-thaw cycle 1**												
E. coli	102.0 ±11.2	152.5 ± 27.2	-	86.6 ± 8.1	99.0 ± 3.6	103.6 ± 4.1	132.7 ± 8.4	99.8 ± 3.1	76.7 ± 0.9	85.5 ± 2.6	112.0 ± 11.4	98.8 ± 1.6
**LLOQ**	124.9 ± 20.7	143.1 ± 20.2	112.6 ± 14.4	119.4 ± 2.4	97.4 ± 1.9	102.2 ± 5.1	163.4 ± 3.8	101.3 ± 3.7	127.8 ± 23.3	105.0 ± 8.7	98.4 ± 6.1	108.9 ± 6.9
**LQC**	140.7 ± 19.5	155.4 ± 13.4	136.0 ± 18.6	129.5 ±22.7	99.0 ± 0.1	96.0 ± 4.0	119.2 ± 5.0	98.5 ± 6.6	105.3 ± 4.0	107.5 ± 8.5	98.3 ± 1.6	99.7 ± 6.2
**MQC**	98.1 ± 16.4	112.1 ± 16.7	61.2 ± 9.9	64.4 ± 0.7	64.9 ± 3.2	78.3 ± 4.8	63.5 ± 7.9	88.2 ± 2.4	71.4 ± 7.2	69.9 ± 12.3	58.6 ± 6.9	65.8 ± 3.9
**HQC**	53.2 ± 1.9	51.6 ±11.3	37.2 ± 3.2	31.9 ± 1.7	26.2 ± 0.7	49.7 ± 2.2	27.7 ± 1.5	51.4 ± 2.6	26.5 ± 1.6	29.3 ± 2.7	31.9 ± 0.8	33.2 ± 1.5
**Freeze-thaw cycle 2**												
E. coli	92.8 ± 12.3	136.7 ± 19.8	-	75.1 ± 2.0	95.4 ± 1.8	96.3 ± 3.1	176.5 ±3.9	94.9 ± 4.0	87.6 ± 6.2	94.3 ± 6.2	116.4 ± 7.5	105.5 ± 1.7
**LLOQ**	116.8 ± 25.4	126.3 ± 5.8	107.3 ± 12.0	108.0 ± 5.6	98.9 ± 3.5	104.6 ± 3.5	216.2 ± 29.1	104.1 ± 2.0	139.9 ± 19.3	110.5 ± 9.3	102.6 ± 10.6	119.3 ± 2.2
**LQC**	128.8 ± 20.4	192.6 ± 35.2	126.9 ± 1.6	107.9 ± 8.8	100.7 ± 6.3	102.6 ± 7.7	139.5 ± 8.0	99.1 ± 2.0	97.9 ± 5.0	111.8 ± 3.7	101.4 ± 6.9	103.9 ± 2.3
**MQC**	71.5 ± 9.5	85.7 ± 7.1	60.3 ± 7.2	55.7 ± 6.6	65.9 ± 0.9	80.9 ± 6.9	73.0 ± 4.1	91.5 ±9.2	68.7 ± 7.3	75.7 ± 3.9	67.0 ± 5.5	72.4 ± 3.6
**HQC**	45.2 ± 3.9	45.2 ± 9.9	30.9 ± 4.0	27.2 ± 2.0	27.8 ± 0.5	48.8 ± 2.3	32.0 ± 1.1	50.4 ± 1.5	25.8 ± 1.2	32.9 ± 2.5	33.6 ± 0.7	36.5 ± 1.2
**Freeze-thaw cycle 3**												
E. coli	132.2 ± 14.8	245.0 ± 72.1	-	76.7 ± 3.5	100.4 ± 9.5	105.2 ± 5.1	213.0 ± 24.5	98.2 ± 5.1	89.7 ± 17.4	88.3 ± 9.0	108.9 ± 2.9	102.9 ± 2.9
**LLOQ**	127.6 ± 10.0	204.5 ± 7.9	97.0 ± 8.9	104.3 ± 6.6	104.9 ± 2.2	102.6 ± 2.1	202.3 ± 18.3	100.4 ±1.2	150.8 ± 19.3	119.4 ± 11.4	106.5 ± 4.5	117.2 ± 2.5
**LQC**	116.1 ± 30.0	176.5 ± 30.1	113.0 ± 11.5	105.1 ± 2.3	94.3 ± 6.2	97.6 ± 2.4	139.4 ± 11.9	99.3 ± 1.9	108.9 ± 4.9	116.3 ± 1.9	100.0 ± 3.1	98.0 ± 5.6
**MQC**	86.4 ± 5.9	115.8 ± 13.6	55.3 ± 2.1	60.8 ± 2.4	65.5 ± 0.5	82.7 ± 4.4	70.7 ± 6.3	96.1 ± 5.2	68.9 ± 5.6	77.0 ± 3.3	62.9 ± 8.4	74.9 ± 3.6
**HQC**	47.8 ± 7.5	60.0 ± 9.8	28.0 ± 2.3	26.0 ± 3.3	26.3 ± 0.5	51.2 ± 1.9	31.9 ± 3.3	52.2 ± 2.7	26.6 ± 1.9	33.6 ± 0.9	32.1 ± 0.7	34.5 ± 3.6
