# Supplementary material for: Zic-HILIC MS/MS Method for NADomics Provides Novel Insights into Redox Homeostasis in Escherichia coli BL21 Under Microaerobic and Anaerobic Conditions
Source: Metabolites. 2024 Nov 9;14(11):607. doi: 10.3390/metabo14110607 (PMC11596675; doi:10.3390/metabo14110607)
Supplement: Supplementary file 1 [file metabolites-14-00607-s001.zip › metabolites-3248822-supplementary_v1/Supplementary table S8_revision_010725.pdf]

Supplementary Table S8. Intracellular concentrations (nmol g-1 CDW) in *E.coli* BL21 quantified by the upgraded zic-HILIC MS/MS method. The data represent absolute metabolite concentrations obtained from one of the biological replicates (n=2) of batch fermenter cultivation with oxygen limitation and anaerobiosis (Figure 2). The table indicates average values from technical replicates (n=5), standard deviation (SD), and relative standard deviation (RSD,%) for each metabolite. Absolute concentrations for NMN were below the limit of quantification.

Sampling Time		NAM	NCA	FAD	NADH	ADPR	NAD^+	NAMN	NADPH	NR	$NADP^+$
T1	Average	8.9	27.1	87.9	253.4	0.4	863.6	43.9	62.8	7.3	238.8
	SD	3.1	5.4	5.3	13.6	0.1	103.9	4.6	4.9	0.4	15.6
	RSD	35.0	20.0	6.0	5.4	15.5	12.0	10.5	7.8	5.2	6.5
T2	Average	14.6	75.9	123.5	285.8	1.2	987.6	54.3	58.3	14.5	171.4
	SD	5.6	20.3	14.7	32.5	0.5	188.7	9.9	4.5	1.6	26.1
	RSD	38.7	26.8	11.9	11.4	41.5	19.1	18.2	7.7	11.0	15.2
T3	Average	14.1	75.4	129.1	284.5	1.9	973.1	50.5	48.4	12.8	159.1
	SD	3.1	17.4	7.9	18.8	0.6	84.9	7.4	1.8	1.7	12.7
	RSD	21.9	23.0	6.1	6.6	31.6	8.7	14.7	3.7	13.4	8.0
T4	Average	10.3	72.3	129.2	260.6	1.4	928.3	45.2	40.6	13.4	142.6
	SD	1.8	24.8	8.4	13.7	0.2	80.6	4.4	4.5	1.8	9.4
	RSD	17.0	34.3	6.5	5.3	15.8	8.7	9.7	11.1	13.0	6.6
T5	Average	16.7	86.8	126.7	260.0	2.5	896.9	46.4	31.8	15.8	132.9
	SD	4.4	26.9	14.3	18.3	0.4	153.3	4.9	2.1	1.7	21.2

**Sampling Time**		**NAM**	**NCA**	**FAD**	**NADH**	**ADPR**	**NAD$^+$**	**NAMN**	**NADPH**	**NR**	**NADP$^+$**
T5	RSD	26.4	31.0	11.3	7.1	13.8	17.1	10.6	6.7	10.7	15.9
